# Sinus of Valsalva aneurysm protruding into the mitral anterior leaflet causing dyspnea: A CARE-compliant case report

**DOI:** 10.1097/MD.0000000000018169

**Published:** 2019-11-27

**Authors:** Baogang Wang, Dashi Ma, Limei Qu, Dianbo Cao, Xiaxia Man

**Affiliations:** a Department of Surgery; b Department of Pathology; c Department of Radiology, the First Hospital of Jilin University, Changchun, China.

**Keywords:** aortotomy, computed tomography angiography, dyspnea, sinus of Valsalva aneurysm, transthoracic echocardiography

## Abstract

**Introduction:**

Sinus of Valsalva aneurysm (SVA) protruding into the mitral anterior leaflet is an extremely rare clinical condition; herein, we present a case of unruptured noncoronary SVA protruding into the mitral anterior leaflet.

**Patient's concerns:**

A 46-year-old male was referred to hospital for exertional dyspnea.

**Diagnosis:**

Transthoracic echocardiography (TTE) and coronary computed tomography angiography (CTA) suggested a noncoronary SVA protruding into the mitral anterior leaflet, causing mitral regurgitation and aortic insufficiency.

**Interventions:**

The aneurysm was resected and the aortic and mitral valves were replaced with mechanical valves via a transaortic approach.

**Outcomes:**

Postoperative recovery was uneventful.

**Conclusions:**

A rare noncoronary SVA protruding into the mitral anterior leaflet can be diagnosed via TTE and CTA. Transaortic mitral surgery is feasible in patients with a dilated aortic annulus ring and mitral valve diseases

## 1 Introduction

Sinus of Valsalva aneurysm (SVA) is a rare clinic entity. Only 0.15% to 1.5% of the cardiopulmonary surgery are done due to SVA.^[1]^ SVA typically occurs in the right sinus (94%), followed by the noncoronary sinus (5%), and the left coronary sinus (less than 1%).^[2]^ Herein we report a case of noncoronary SVA protruding into the anterior leaflet of the mitral valve. The ethics committee of the Jilin University First Hospital had approved the study and the publication of the case report. Informed consent was obtained from the patient.

## 2 Case presentation

**Figure 1:**
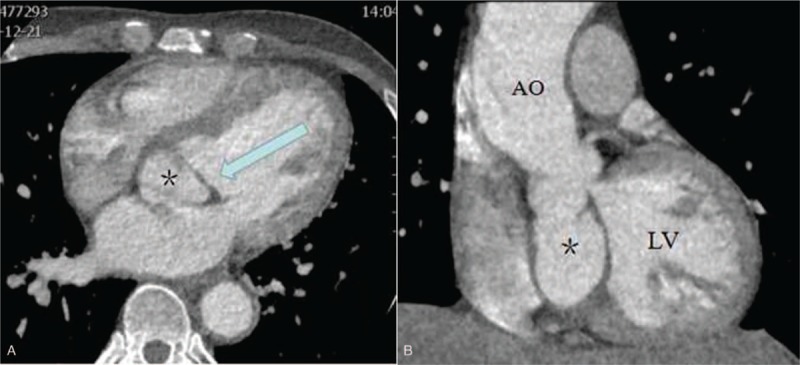
(A) Sinus of Valsalva aneurysm protruding into mitral anterior leaflet. ∗ is sinus of Valsalva aneurysm, the blue arrow is the mitral anterior leaflet. (B) The sac of sinus of Valsalva aneurysm was measured 36 × 47 × 51 mm. ∗ is the sac of aneurysm. AO = ascending aorta, LV = left ventricle.

A 46-year-old male (weight 61 kg, height 170 cm) was admitted for exertional dyspnea. There was no history of fever. Physical examination revealed a systolic mitral murmur and a diastolic aortic murmur. No dyskinesia or paresthesia was detected. Transthoracic echocardiography (TTE) revealed a noncoronary SVA projecting into the mitral anterior leaflet, leading to moderate mitral regurgitation and severe aortic insufficiency, combined with a dilated aortic root. The ascending aorta measured 54-mm, and the left ventricular diastolic diameter was 75-mm, with a left ventricular ejection fraction of 66%. No ventricular septal defect was detected. Coronary computed tomography angiography (CTA) confirmed that there were no abnormalities or stenoses found in the coronary tree, and that the sac of the noncoronary SVA (measuring 36 × 47 × 51 mm) was protruding into the anterior leaflet of the mitral valve (Fig. 1A and B).

**Figure 2:**
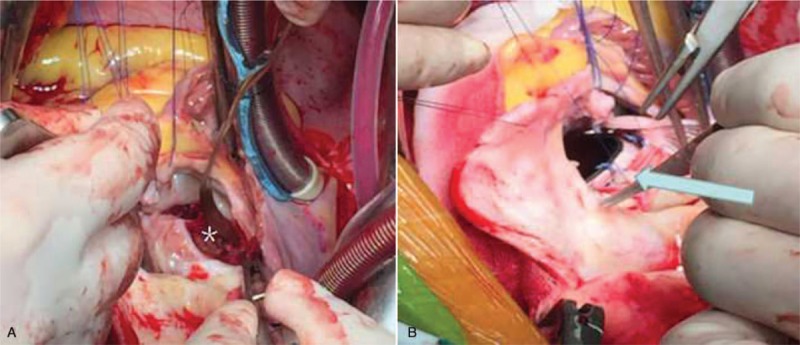
(A) Removal of the thrombus in the sac of SVA intraoperatively. ∗ is the thrombus. (B) Performing the mitral replacement via a transaortic approach, the blue arrow is the mitral mechanical valve. SVA = sinus of Valsalva aneurysm.

Surgery was performed via median sternotomy. Considering the marked dilation of the ascending aorta, we established cardiopulmonary bypass via right formal artery-bicaval cannulation. The ascending aorta was not adhered to the pericardium, and was clamped proximal to the dilated part. Close examination of the tricuspid aortic valve and ascending aortic wall revealed the thickening of the wall, indicating inflammation. The unruptured sac of the noncoronary SVA was protruding downwards into the body of the mitral anterior leaflet, and contained a thrombus mass. After excision of the tricuspid mitral valve and the aneurysm and removal of the thrombus (Fig. 2A), the mitral valve apparatus could be clearly observed through the aortic annulus. Mitral valve replacement was performed via the transaortic approach. We excised the anterior and posterior leaflets of the mitral valve, and reconstructed the mitral annulus with a patch (Chest.Co, Shanghai, China). A Sorin mechanical valve (27 mm, Sorin Group, Italia) was implanted using interrupted mattress sutures (Fig. 2B). The aortic valve was replaced with a Sorin mechanical valve (23 mm, Sorin Group, Italia), and the ascending aorta was replaced with an artificial vessel (IGW0028-15, La Ciotat, France). The proximal end of the artificial vessel was located 5 mm above the ostium of the right coronary artery, and the distal end was located at the proximal part of the brachiocephalic trunk artery. The artificial vessel was enveloped by the intrinsic aortic wall.

**Figure 3:**
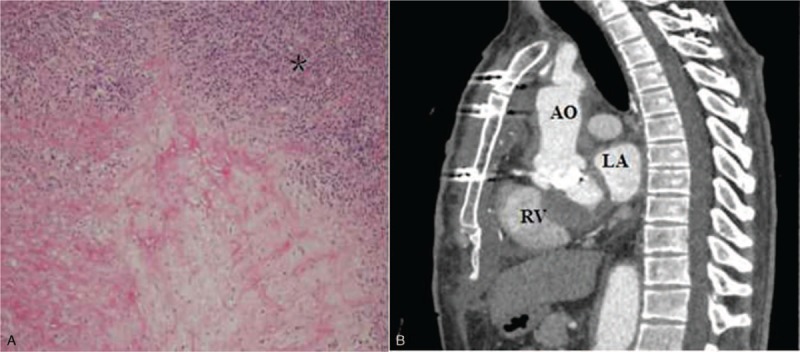
(A) Histopathological examination revealed the infiltration of inflammatory cells in the mitral valve. ∗ is the inflammatory cells (HE staining, ×100). (B) Restoration of the anatomy of the aortic root postoperatively. AO = ascending aorta, HE = hematoxylin eosin, LV = left ventricle, RV = right ventricle.

Pathological examination of the aortic and mitral valves using hematoxylin/eosin staining demonstrated that mucoid degeneration, abscess formation, and substantial infiltration of inflammatory cells (Fig. 3A). Antibiotics were administered for 5 weeks. The postoperative course was uneventful. Follow-up TTE showed satisfactory functioning of both replaced valves. Aortic CTA demonstrated the restoration of the normal anatomical relationship at the aortic root (Fig. 3B).

## 3 Discussion

SVA can be congenital or acquired. Congenital SVA is caused by localized weakness of the elastic lamina at the junction of the aortic media and fibrotic annulus; this is usually associated with other abnormalities, such as ventricular septal defect and congenital connective tissue diseases, including Marfan syndrome and Ehlers–Danlos syndrome. The etiologies of acquired SVA include degenerative diseases, thoracic trauma, atherosclerosis, cystic medial necrosis, and infective endocarditis. Unruptured SVA is typically asymptomatic. The symptoms of ruptured SVA may vary, including clinical silence, decrease in exertional endurance, aortic insufficiency, disturbance of cardiac conductance, and cardiogenic shock.

To our knowledge, noncoronary SVA protruding into the mitral anterior leaflet has not been previously described in literature. The present patient did not have a history of fever or exhibit any remarkable signs of endocarditis preoperatively. However, histopathological examination revealed that neutrophil infiltration in the resected specimens, suggesting that the underlying cause of SVA may have been latent infective endocarditis. The high pressure in the ascending aorta caused the aneurysm to project through the weakened aortic annulus into the body of the mitral anterior leaflet. The blood entering the blind-ended sac of the unruptured aneurysm then gradually formed a thrombus. The secondary mitral regurgitation and aortic insufficiency caused exertional dyspnea and left ventricular dilation.

SVA is diagnosed using TTE and transesophageal echocardiography (TEE). The major advantage of TEE is the proximity of the probe to the aortic root, enabling better examination of the anatomy of the aneurysm and adjacent tissues.^[3]^ TTE and TEE also provide information on left ventricular function, and can identify the volume of the left-to-right shunt in cases of involving a ruptured aneurysm. CTA and cardiac magnetic resonance imaging have become useful tools; the methods can reveal the size and shape of the aneurysm, obviating the need for preoperative catheterization in surgical planning.^[4]^

The main treatment of SVA is surgery. However, due to the rarity of SVA, there are no clear guidelines for surgical treatment of SVA. The presence of a ruptured aneurysm usually increases the biventricular volume overload, and should be corrected as soon as possible. The decision to intervene in cases of asymptomatic, unruptured SVA depends mainly on the aneurysm size and the involvement of adjacent structures.^[5]^ The main aim of surgery is to reconstruct the continuity of the aortic wall, and close the connection between the aneurysm and other cardiac chambers. A decision must be made intraoperatively on whether to repair or replace the involved valves, according to the extent of valvular regurgitation. Coexistent intracardiac abnormalities must also be corrected. Our patient had aortic insufficiency combined with mitral regurgitation. The ascending aorta measured 55 mm, and the diameter of the aortic sinus was 50 mm. Therefore, we performed mitral valve replacement via a transverse aortotomy approach; this approach was first reported by Carmichael et al^[6]^ (1983) for mitral valve replacement or repair.^[7]^ The transverse aortotomy approach has also been used in the treatment of Marfan's syndrome,^[8]^ acute infective endocarditis,^[9]^ and even mitral chordae-preserving bivalvular replacement.^[10]^ Liu et al^[7]^ reported that transverse aortotomy for mitral valve replacement via was superior to the transatrial septal approach regarding operation time, cardiac injury, exposure of the mitral valve, and redo surgery. The indication for the transverse aortotomy approach is dilation of the aortic annulus combined with a mitral valve lesion, but without other intracardiac abnormalities. Due to the rarity of literature about this approach, there have been no conclusions as to how wide the aortic annulus must be for the transaortic approach. Wang^[11]^ suggests that the transaortic approach can be applied as long as the surgical instruments and artificial prosthetic valve can fit through the aortic annulus. The key to applying this technique is the clear exposure of the mitral valve. The shortcoming of this approach is that we cannot retreat the mitral valve via the same transaortic approach if TEE reveals unsatisfactory mitral valve repair. Hence, we established cardiopulmonary bypass so that the mitral regurgitation could be treated via the transatrial septum approach if necessary.

## Acknowledgments

We thank Kelly Zammit, BVSc, from Liwen Bianji, Edanz Group China (www.liwenbianji.cn/ac), for editing the English text of a draft of this manuscript.

## Author contributions

**Conceptualization:** Baogang Wang.

**Data curation:** Baogang Wang, Dianbo Cao.

**Funding acquisition:** Xiaxia Man.

**Investigation:** Baogang Wang, Dashi Ma, Limei Qu, Xiaxia Man.

**Methodology:** Dashi Ma, Limei Qu, Dianbo Cao, Xiaxia Man.

**Resources:** Dianbo Cao.

**Supervision:** Dashi Ma, Limei Qu.

**Validation:** Baogang Wang.

**Writing – original draft:** Baogang Wang.

**Writing – review & editing:** Xiaxia Man.
